# Erythema Multiforme: A Presentation of COVID-19 Pneumonia

**DOI:** 10.7759/cureus.26835

**Published:** 2022-07-14

**Authors:** Megha Puri, William A Vasquez Espinosa, Mary Tobiasson, Chirag Patel

**Affiliations:** 1 Internal Medicine, Saint Peter’s University Hospital, New Brunswick, USA

**Keywords:** covid-19, generalized rash, target sign, erythema multiforme major, organizing pneumonia

## Abstract

Since its emergence in December 2019, coronavirus disease 2019 (COVID-19) has been detrimental worldwide. Although COVID-19 infection is primarily known for its respiratory manifestations, extrapulmonary features are increasingly being reported. Among these, cutaneous manifestations are apparent but have a high likelihood of not being attributed to COVID-19. We present the case of a 63-year-old female unvaccinated against COVID-19. She presented with fever, cough, shortness of breath, and rash. The symptoms were present for four days and appeared after contact with a confirmed symptomatic COVID-19-positive family member. The rash started first on the chest and then spread to the face and whole body including the palms and soles. It was erythematous and maculopapular and is associated with ulceration and swelling of the lips. In places, it was confluent and had a target-like appearance. On admission, the severe acute respiratory syndrome coronavirus 2 (SARS-CoV-2) polymerase chain reaction (PCR) was negative. As she was septic with initial suspicion of tick-borne infections, she was started on doxycycline. Given her symptoms on presentation, the suspicion of COVID-19 was very high, and the SARS-CoV-2 nasal swab PCR was repeated, which was negative yet again. With the index of suspicion being very high, her presentation was speculated to be atypical, especially in the setting of a target-like rash involving the palms and soles. The antibody was checked. IgG antibodies for SARS-CoV-2 were positive. All other antibodies for mycoplasma, Lyme disease, *Ehrlichia*, and Rocky Mountain spotted fever (RMSF) were negative. Parvovirus DNA and chikungunya IgG, antinuclear antibody (ANA), and antineutrophil cytoplasmic antibody (ANCA) screens were negative. IgG for mycoplasma, dengue, and herpes simplex virus 1 (HSV1) were positive. During all this time, the patient did not show clinical improvement in spite of being on antibiotics. In fact, her oxygen saturation dropped, and she required oxygen through the nasal cannula. A lung tissue biopsy taken on bronchoscopy showed chronic inflammation and organizing pneumonia. To note, mycoplasma DNA PCR and HSV culture from bronchoalveolar lavage (BAL) were negative.

The patient was started on intravenous steroids. A confirmatory skin biopsy was done, and it showed perivascular, interstitial, and spongiotic dermatitis related to a viral infection. While on steroids, the patient improved dramatically. Her skin rash also improved, and she was discharged. On outpatient follow-up, she was doing exceptionally well with ambulatory oxygen saturation of 100%.

This patient who was COVID-19 PCR-negative twice could have been easily deemed as not having COVID-19. However, the fact that she was unvaccinated, had positive sick contact with imaging concern for COVID-19 pneumonia, and COVID-19 antibody being positive and no other test being positive clearly attributes her manifestations to the virus. The presence of a rash could easily be misleading. Awareness of the fact that a rash like erythema multiforme (EM) could be a sign of underlying COVID-19 is extremely prudent and is an addition to the ever-expanding knowledge of this virus.

## Introduction

Since the time it first emerged in December 2019, coronavirus disease 2019 (COVID-19) has been detrimental worldwide. With constant undergoing research, it still is an ocean to be explored in-depth. Although COVID-19 infection is primarily known for its respiratory manifestations, extrapulmonary features including cutaneous manifestations are increasingly being reported [1]. Among the various features, cutaneous manifestations are very much apparent and have a high likelihood of not being attributed to COVID-19. Furthermore, the relationship between the various extrapulmonary manifestations and the severity and prognosis of the COVID-19 disease need further studies to be established.

## Case presentation

**Figure 1:**
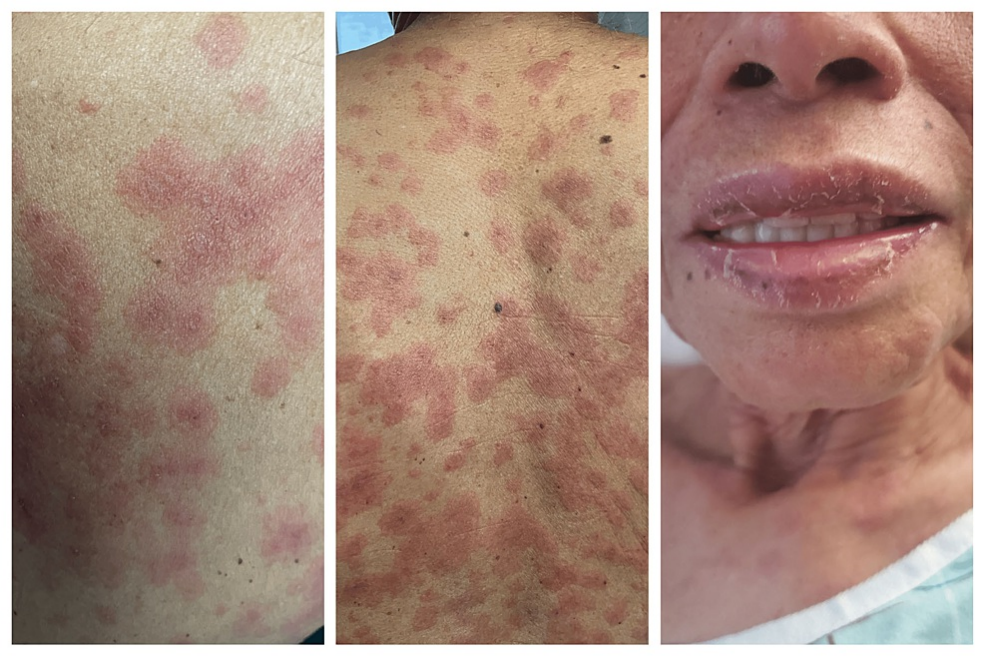
Target-like lesions with tongue ulcerations

A 63-year-old female unvaccinated against COVID-19 was admitted with complaints of fatigue, cough, mild shortness of breath, fever, and a rash. The symptoms started after the patient had contact with her daughter who was a confirmed symptomatic COVID-19 patient. As per the patient, the rash started four days prior to her presentation to the hospital. It started first on the chest and then spread to the face and involved the whole body including the palms and soles. On examination, the rash was erythematous and maculopapular, diffused all over the body, and was associated with ulceration and swelling of the lips (Figure 1).

In places, it was confluent and had a target-like appearance consistent with a rash similar to erythema multiforme (EM). At the time she was admitted, she was found to be tachycardic with a heart rate of 121 bpm but not tachypneic and saturating above 95% on room air. Basic bloodwork showed leukocytosis and lactic acidosis. As the patient was in sepsis, she was started on IV fluids and antibiotics, ceftriaxone, and also doxycycline to cover for possible tick-borne infections. The rest of the admission laboratory results showed elevated inflammatory markers including erythrocyte sedimentation rate, C-reactive protein, and D-dimer (1,635 ng/mL). Hence, a computed tomography pulmonary angiogram (CTPA) was done that did not show pulmonary embolism. However, it showed bilateral peripheral opacities consistent with multifocal pneumonia with concern for COVID-19 pneumonia. Ironically, severe acute respiratory syndrome coronavirus 2 (SARS-CoV-2) nasal swab polymerase chain reaction (PCR) done on admission was negative. As the patient had a history of recent close COVID-19 contact and CTPA concerning COVID-19 pneumonia, the suspicion of COVID-19 was high. A repeat SARS-CoV-2 testing was done, and it was negative again. It should also be noted here that the patient showed no clinical improvement with the antibiotics. Due to consistently high suspicion of COVID-19 organizing pneumonia, the antibody was checked. IgG antibodies for SARS-CoV-2 were positive. The other antibodies checked were mycoplasma, Lyme disease, *Ehrlichia*, and Rocky Mountain spotted fever (RMSF), which were negative, as well as antinuclear antibody (ANA) and antineutrophil cytoplasmic antibody (ANCA) screens. Parvovirus DNA and chikungunya IgG were also negative, while IgG for mycoplasma, dengue, and HSV1 were positive. The working diagnosis at this time was COVID-19 pneumonia versus mycoplasma. The patient was given levofloxacin and vancomycin, which were changed to linezolid as there were no signs of improvement in the patient’s status. Despite being on multiple antibiotics, the patient did not show any improvement; in fact, her oxygen saturation dropped to the point that she required oxygen supplementation of 2-3 L with a nasal cannula. At this time, a bronchoscopy was done, and a sample for bronchoalveolar lavage (BAL) was taken. Mycoplasma DNA PCR and HSV culture from BAL was negative. A lung tissue biopsy showed chronic inflammation and organizing pneumonia.

**Figure 2:**
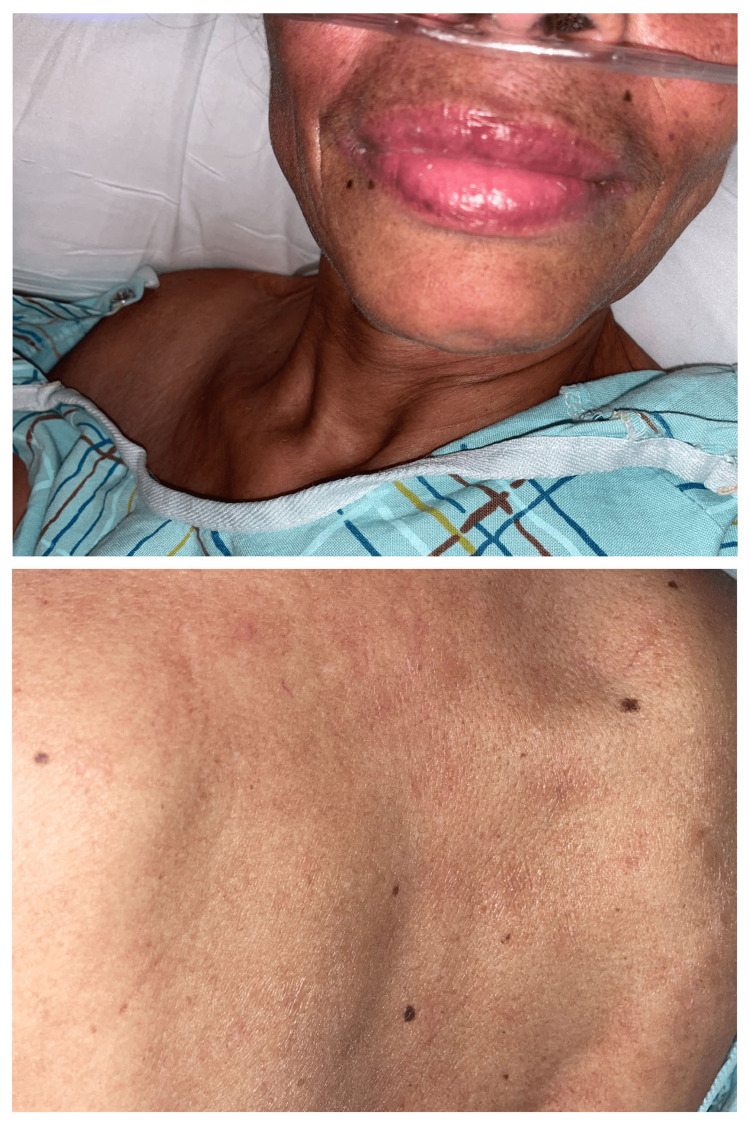
Resolution of the erythema multiforme rash

Hence, the patient was started on intravenous steroids. While on steroids, the patient showed dramatic improvement. The requirement for oxygen supplementation decreased, and eventually, the patient was on room air. Clinically, she felt better. The EM-like rash improved on initiation of steroids and resolved completely before the patient was discharged (Figure 2).

A skin biopsy was done from one of the sites of rash, and it showed perivascular, interstitial, and spongiotic dermatitis related to a viral infection. After discharge, the patient was followed as an outpatient, and she was doing exceptionally well with ambulatory oxygen saturation of 100%.

## Discussion

Erythema multiforme is an immune-mediated mucocutaneous condition. The lesions are typically targetoid-like with concentric variation in color. Various causative factors are attributed to the development of EM, which include mainly infections, medications, immunization, underlying malignancy, autoimmune disease, radiation, and menstruation [2,3]. Herpes simplex virus (HSV) and *Mycoplasma pneumoniae* are the main infectious agents, but other viruses have been reported, such as adenovirus, coxsackieviruses, and parvovirus B19.

New information, especially extrapulmonary manifestations, including cutaneous presentations of COVID-19, is emerging every day [4,5]. We suggest that EM or EM-like rash could be another pattern of cutaneous manifestation of COVID-19. There have been a few case reports of EM being seen with COVID-19; however, in most of those cases, the patient was also getting a number of medications that could be the causative factor in the development of EM rash [6,7]. This particular patient did not report taking any medication before the onset of the rash and hence can be said to have a more direct causal relationship of COVID-19 with EM-like rash.

Erythema multiforme in COVID-19 patients is attributed to a delayed hypersensitivity reaction. It has been seen usually in patients with age less than 30 years or older patients with age more than 55 years, or it could be a drug-induced reaction [6].

Our patient, who did not have typical COVID-19 pneumonia symptoms initially and was SARS-CoV-2 PCR-negative twice, could have been easily deemed as not having COVID-19. Her only presentation to the hospital was fever and rash. However, the fact that she was unvaccinated and had positive sick contact with imaging concern of COVID-19 pneumonia and COVID-19 antibody being positive clearly attributes her manifestations to the virus. The other tests done to look for a causative factor were negative. She had not received any medications before she started having the EM-like rash that her symptoms could be attributed to. Our patient had both cutaneous and oral mucosa with lesions. Hence, this EM-like rash is highly likely to be erythema multiforme major.

## Conclusions

COVID-19 is still an ocean to be explored. The focus is not only on pulmonary signs and symptoms but also on multisystemic features taken all together that this virus can present. The fact that a rash like EM could be associated with COVID-19 is extremely prudent. Not many cases have been reported, and further studies are required to establish a confirmed relationship between EM and COVID-19. The future effects of COVID-19 are still unknown. EM being attributed as a delayed hypersensitivity reaction could open new doors to the study of various other immunological reactions that the virus could bring about. It could give us more insight into the pathophysiology of the disease and eventually help enhance the current pharmacological interventions.

In addition to the pulmonary and cutaneous features, a high level of vigilance is required to catch other extrapulmonary manifestations of the COVID-19 disease as well. The ultimate goal is to collaborate data obtained worldwide to build up solid literature on this virus to be prepared for the future effects, if any, of the COVID-19.

## References

[1] Genovese G, Moltrasio C, Berti E, Marzano AV (2021). Skin manifestations associated with COVID-19: current knowledge and future perspectives. Dermatology.

[2] Huff JC, Weston WL, Tonnesen MG (1983). Erythema multiforme: a critical review of characteristics, diagnostic criteria, and causes. J Am Acad Dermatol.

[3] Sokumbi O, Wetter DA (2012). Clinical features, diagnosis, and treatment of erythema multiforme: a review for the practicing dermatologist. Int J Dermatol.

[4] Matar S, Oulès B, Sohier P, Chosidow O, Beylot-Barry M, Dupin N, Aractingi S (2020). Cutaneous manifestations in SARS-CoV-2 infection (COVID-19): a French experience and a systematic review of the literature. J Eur Acad Dermatol Venereol.

[5] Daneshgaran G, Dubin DP, Gould DJ (2020). Cutaneous manifestations of Covid-19: an evidence-based review. Am J Clin Dermatol.

[6] Bennardo L, Nisticò SP, Dastoli S (2021). Erythema multiforme and COVID-19: what do we know?. Medicina (Kaunas).

[7] Jimenez-Cauhe J, Ortega-Quijano D, Carretero-Barrio I, Suarez-Valle A, Saceda-Corralo D, Moreno-Garcia Del Real C, Fernandez-Nieto D (2020). Erythema multiforme-like eruption in patients with COVID-19 infection: clinical and histological findings. Clin Exp Dermatol.

